# Shiga Toxin-Producing *Escherichia coli* in Plateau Pika (*Ochotona curzoniae*) on the Qinghai-Tibetan Plateau, China

**DOI:** 10.3389/fmicb.2016.00375

**Published:** 2016-03-22

**Authors:** Xiangning Bai, Wang Zhang, Xinyuan Tang, Youquan Xin, Yanmei Xu, Hui Sun, Xuelian Luo, Ji Pu, Jianguo Xu, Yanwen Xiong, Shan Lu

**Affiliations:** ^1^State Key Laboratory for Infectious Disease Prevention and Control, National Institute for Communicable Disease Control and Prevention, Chinese Center for Disease Control and PreventionBeijing, China; ^2^Qinghai Institute for Endemic Disease Prevention and ControlXining, China; ^3^Collaborative Innovation Center for Diagnosis and Treatment of Infectious DiseasesHangzhou, China

**Keywords:** Shiga toxin-producing *Escherichia coli*, plateau pika, MLST, PFGE, antibiotic resistance

## Abstract

Shiga toxin-producing *Escherichia coli* (STEC) are an emerging group of zoonotic pathogens. Ruminants are the natural reservoir of STEC. In this study we determined the prevalence and characteristics of the STEC in plateau pika (*Ochotona curzoniae*) on the Qinghai-Tibetan Plateau, China. A total of 1116 pika samples, including 294 intestinal contents samples, 317 fecal samples, and 505 intestinal contents samples, were collected from May to August in the years 2012, 2013, and 2015, respectively. Twenty-one samples (1.88%) yielded at least one STEC isolate; in total, 22 STEC isolates were recovered. Thirteen different O serogroups and 14 serotypes were identified. One *stx*_1_ subtype (*stx*_1a_) and three *stx*_2_ subtypes (*stx*_2a_, *stx*_2b_, and *stx*_2d_) were present in the STEC isolates. Fifteen, fourteen, and three STEC isolates harbored the virulence genes *ehxA*, *subA*, and *astA*, respectively. Adherence-associated genes *iha* and *saa* were, respectively, present in 72.73 and 68.18% of the STEC isolates. Twenty antibiotics were active against all the STEC isolates; all strains were resistant to penicillin G, and some to cephalothin or streptomycin. The 22 STEC isolates were divided into 16 pulsed-field gel electrophoresis patterns and 12 sequence types. Plateau pikas may play a role in the ongoing circulation of STEC in the Qinghai-Tibetan plateau. This study provides the first report on STEC in plateau pikas and new information about STEC reservoirs in wildlife. Based on the serotypes, virulence gene profiles and multi-locus sequence typing (MLST) analysis, the majority of these pika STECs may pose a low public health risk.

## Introduction

Shiga toxin-producing *Escherichia coli* (STEC) represents an emerging group of zoonotic pathogens causing diarrhea, hemorrhagic colitis (HC), and the life-threatening hemolytic uremic syndrome (HUS) in humans (Smith et al., 2014). Strains of O157 serogroup have been considered to be most virulent, and STEC O157 strains have been extensively studied and shown to be involved in many cases and outbreaks of human disease (Doorduyn et al., 2006; Friesema et al., 2007; Sigmundsdottir et al., 2007; Greenland et al., 2009). However, there is growing concern over the emergence of more than 200 non-O157 STEC serotypes associated with human illness, including the life threatening HUS (Coombes et al., 2008). In fact, non-O157 strains are responsible for a larger portion of STEC infections than O157 strains in the United States, Canada, Australia, Latin America, and Europe (Karmali et al., 1985; Caprioli et al., 1997; Tozzi et al., 2003; Brooks et al., 2005). At present, detection methods and in depth studies are mainly focused on O157:H7 and the top six serogroups of STEC (O26, O45, O103, O111, O121, and O145) (Conrad et al., 2014; Wasilenko et al., 2014), thus the public health significance of STEC of other serotypes is likely to be underestimated due to the high diversity of genotypes and phenotypes.

Shiga toxin (Stx) is the most critical virulence factor of STEC, consisting of two broad immunologically different types: Stx1 with three subtypes (Stx1a, Stx1c, and Stx1d) and Stx2 with seven subtypes (Stx2a to Stx2g). Stx can damage intestinal epithelial cells and kidneys, causing HC and HUS (Smith et al., 2014). However, many non-O157 strains that produce Shiga toxin have been not associated with HUS cases, indicating additional virulence determinants may play a role in pathogenesis. Following initial attachment of STEC to host intestinal cells, strains that express intimin can intimately attach to host cells and cause the attaching-and-effacing lesion (Frankel and Phillips, 2008). Intimin is encoded by *eae* that resides on a chromosomal pathogenicity island called the locus of enterocyte effacement (LEE). Enterohemolysin (EhxA), encoded by a 60-MDa virulence plasmid in some STEC strains, that readily causes hemolysis of washed sheep erythrocytes, is another important virulence factor that contributes to severe disease in humans (Cookson et al., 2007). A number of other adherence structures and virulence factors have also been proposed to contribute to STEC pathogenesis (Coombes et al., 2008; Tseng et al., 2014; Chui et al., 2015).

Though the main reservoir of STEC is the intestinal tract of cattle, many other domestic and wild animals including pig, sheep, dog, deer, wild boar, and hare are also sources of STEC (Oporto et al., 2008; Miko et al., 2009; Sanchez et al., 2010; Bentancor et al., 2012; Shen et al., 2015). STEC strains have been described recently in diarrheal patients, domestic animals and foodstuffs of animal origin in China (Xiong et al., 2012; Chen et al., 2014; Meng et al., 2014; Bai et al., 2015; Wang et al., 2015; Yu et al., 2015), but little data is available concerning wildlife. The plateau pikas (*Ochotona curzoniae*) are underground-dwelling mammals (order Lagomorpha) mostly living in the extremely harsh wild environments of severe cold, low atmospheric oxygen and strong ultraviolet radiation of the Qinghai-Tibetan plateau, China. Plateau pikas have close contact with yaks. In our previous study, we explored the occurrence and characteristics of STEC in yaks (*Bos grunniens*) for the first time, revealing that yaks are natural reservoirs of STEC, and some of the STEC strains they harbored had the potential to cause human disease (Bai et al., 2013). In this study, we investigated the prevalence of STEC in plateau pikas in this unique ecosystem.

## Materials and Methods

### Collection of Samples

Four sites were enrolled in this study in Yushu tibetan autonomous prefecture, Qinghai province, China, including Guoqinggou [3,929 m above msl (mean sea level), latitude of 33°7′ and longitude of 96°84′), Jielachong (3,970 m above msl, latitude of 33°48′ and longitude of 96°51′), Gandacun (4,322 m above msl, latitude of 33°13′ and longitude of 96°73′), Batangtan (3,987 m above msl, latitude of 32°51′ and longitude of 96°56′)]. The plateau pikas were captured by ‘mousetrap’ in their breeding seasons from May to August when their population densities are high, and the colon contents were collected. Fresh feces were picked up from the holes. In total, 1116 samples were collected consisting of 294 colon contents samples, 317 fecal samples, and 505 colon contents samples in the years 2012, 2013, and 2015, respectively. Samples were mixed in Luria-Bertani medium containing 30% glycerol (Land Bridge, Beijing, China), stored at -20°C immediately, and transported to the laboratory in National Institute for Communicable Disease Control and Prevention, China CDC, in ice cold conditions for the screening of STEC.

### Isolation of STEC

One gram of each sample was enriched into 5 ml of *E. coli* broth (Land Bridge, Beijing, China) and incubated at 37°C for 18 to 24 h with shaking at 200 rpm. The enriched samples were investigated for *stx*_1_ and *stx*_2_ genes by duplex PCR assay as used in our previous study (Bai et al., 2015). Briefly, 1.5 ml of each enrichment sample was centrifuged at 13,000 × *g* for 2 min, the pellet was suspended in 150 μl of lysis buffer (100 mM NaCl, 10 mM Tris-HCl [pH 8.3], 1 mM EDTA [pH 9.0], 1% Triton X-100), then boiled for 10 min, and centrifuged at 13,000 × *g* for 2 min. The supernatant was then used as the PCR template. Enriched samples that tested positive for *stx*_1_ and/or *stx*_2_ genes were plated onto CHROMagar^TM^ ECC agar (CHROMagar, Paris, France), and MacConkey agar (Oxoid, Hampshire, UK) and then incubated at 37°C overnight. About 10 colonies (blue or colorless, round moist *E. coli*-like colonies) on CHROMagar^TM^ ECC and 5 pink or red colonies on MacConkey were picked to screened for the presence of *stx*_1_ and/or *stx*_2_ genes by single colony duplex PCR assay (Bai et al., 2015). If all colonies were negative for *stx*, another 10 and 5 colonies on the two plates were picked and screened. Finally, with the exception of different colony colors (blue or colorless), *stx* types or *stx* combinations present in the same sample, only one STEC isolate from each *stx*-positive sample was kept for further identification as described previously (Bai et al., 2015).

### Biochemical Tests and Serotyping of STEC Isolates

Each *stx*-positive isolate was confirmed to be *E. coli* by biochemical identification using the API 20E system (bioMérieux, Marcy l’Etoile, France). The O serogroup was determined using all available *E. coli* antisera, i.e., O1–O188 (Statens Serum Institut, Hillerød, Denmark). The entire coding sequence of *fliC* was amplified by PCR using primers F-FLIC1 (5′-ATGGCACAAGTCATTAATACCCAAC-3′) and R-FLIC2 (5′-CTAACCCTGCAGCAGAGACA-3′), as reported by Fields et al. (1997), then sequenced and compared to a publicly available CGE SerotypeFinder web tool^1^ to determine the H type of each isolate (Joensen et al., 2015).

### *stx* Subtyping

The *stx*_1_ subtypes of STEC isolates were determined by a PCR-based subtyping method devised by Scheutz et al. (2012). The complete *stx*_2_ gene was amplified as described by Gunzer et al. (1992), then cloned into vector pMD18-T and transformed into *E. coli* JM109 (Takara, Dalian, China). About 10 transformants were selected for sequencing to discern multiple *stx*_2_ subtypes in a PCR product. A neighbor-joining tree was constructed using MEGA 6 (Tamura et al., 2013) to assign the *stx*_2_ subtype (Scheutz et al., 2012).

All *stx*_2_ nucleotide sequences determined in this study have been submitted to GenBank under accession numbers KU158844-KU158861.

### Detection of Virulence and Adherence Factors

Shiga toxin-producing *Escherichia coli* isolates were subjected to PCR for detection of intimin-encoding gene (*eae*), putative adhesin genes (*iha*, *efa1*, *saa*, *paa*, *eibG*), virulence-associated genes (*ehxA*, *katP*, *toxB*, *astA*, *subA*), the high-pathogenicity island (HPI) marker genes (*irp2* and *fyuA*) and 11 non-LEE encoded effector (*nle*) genes (*ent*, *nleA*, *nleB*, *nleB2*, *nleE*, *nleH1-1*, *nleH1-2*, *nleC*, *nleD*, *nleF*, *nleG*) using primers listed in Supplementary Table S1.

### Antimicrobial Susceptibility Testing

Antimicrobial drug susceptibility was determined by the disk diffusion method as recommended by the Clinical and Laboratory Standards Institute (Clinical Laboratory Standards Institute [CLSI], 2014). We tested the following 23 antimicrobial agents: penicillin G, amoxicillin/clavulanic acid, ampicillin/sulbactam, levofloxacin, nitrofurantoin, aztreonam, norfloxacin, chloramphenicol, cefepime, cephalothin, meropenem, ceftria xone, imipenem, streptomycin, ciprofloxacin, gentamicin, piperacillin, cefotaxime, nalidixic acid, kanamycin, trimethoprim-sulfamethoxazole, cefuroxime, and tetracycline (Oxoid, Hampshire, UK). Results were used to classify isolates as being resistant or susceptible to a particular antibiotic using standard reference values (Clinical Laboratory Standards Institute [CLSI], 2014).

### Pulsed-Field Gel Electrophoresis (PFGE)

Shiga toxin-producing *Escherichia coli* isolates were digested with *Xba*I and separated by pulsed-field gel electrophoresis (PFGE) according to the protocol for non-O157 STEC from PulseNet, USA^2^. Gel images were captured with a Gel Doc^TM^ XR+ system (Bio-Rad, Hercules, CA, USA). An UPGMA (unweighted pair-group method with arithmetic mean) dendrogram was constructed using BioNumerics software version 4.0 (Applied Maths, Sint-Martens-Latem, Belgium).

### Multi-locus Sequence Typing (MLST)

All STEC isolates were analyzed by multi-locus sequence typing (MLST) according to the *E. coli* MLST website^3^. Sequences types (STs) of the HUSEC (HUS-associated enterohemorrhagic *E. coli*) collection were obtained from http://campus.uni-muenster.de/hyg_klhus_husec.html?&L~=~1 (Mellmann et al., 2008). All human STEC STs of O157 and the top six serogroups were obtained from the *E. coli* MLST website. A minimum spanning tree based on these STs was generated using BioNumerics software.

### Statistical Analysis

The χ^2^ test was performed using SAS software version 9.1 (SAS Institute Inc., Cary, NC, USA). *P* < 0.05 was considered statistically significant.

### Ethics Statement

The pikas in this study were captured by ‘mousetrap’ by local center for disease control and prevention for communicable diseases surveillance. The study was approved by the ethics committee of National Institute for Communicable Disease Control and Prevention, China CDC, according to the medical research regulations of National Health and Family Planning Commission of the People’s Republic of China.

## Results

### Prevalence of STEC in Plateau Pika Samples

Twenty-two STEC isolates were obtained from 21 of the 58 *stx*-positive plateau pika samples giving a culture positive rate of 36.21% for *stx*-positive samples and 1.88% for all samples (**Table 1**). Single isolate was obtained from 19 intestinal content samples and one fecal sample. Two isolates (15ST443 and 15ST444) with different *stx* types were recovered from one intestinal contents sample collected in 2015. Nine out of 294 intestinal contents samples collected in 2012, one out of 317 fecal samples collected in 2013, and 11 out of 505 intestinal contents samples yielded STEC isolates, giving culture positive rates of 3.06, 0.32, and 2.18% for all samples, respectively (**Table 1**). The culture positive rates in intestinal contents were much higher than that in feces (*P* < 0.05).

**Table 1:** Prevalence of Shiga toxin-producing *Escherichia coli* in plateau pika.

Year	Type of sample	Number of samples	Number of *stx* positive (%)	Number of samples with STEC isolates (%)	Number of STEC isolates (%)
2012	Intestine contents	294	10 (3.40)	9 (3.06)	9 (3.06)
2013	Feces	317	2 (0.63)	1 (0.32)	1 (0.32)
2015	Intestine contents	505	46 (9.11)	11 (2.18)	12 (2.38)
Total		1116	58 (5.20)	21 (1.88)	22 (1.99)

### Serogroups and Serotypes

**FIGURE 1:**
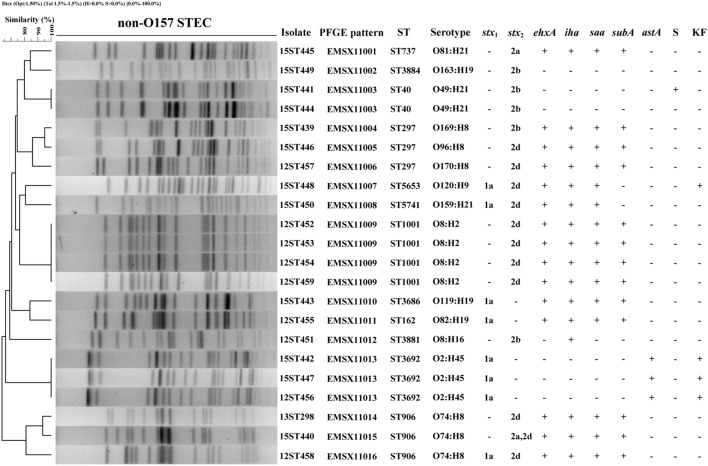
**Pulsed-field gel electrophoresis (PFGE) profiles of 22 non-O157 Shiga toxin-producing *Escherichia coli* (STEC) isolates from plateau pikas.** The corresponding isolate names, PFGE patterns, sequence types (STs), serotypes, *stx*_1_ and *stx*_2_ subtypes, the profiles of *ehxA*, *iha*, *saa*, *subA*, and *astA* genes, and antibiotic resistances are listed on the right. Year of sampling is contained in the first two numbers of the isolate names: 12 means year 2012, 13 means 2013, and 15 means 2015. Abbreviations for antibiotics are: S, Streptomycin; KF, Cephalothin. ‘+’ means genes positive or antibiotic resistant; ‘-’ means genes negative or antibiotic susceptible.

In total, 13 different O serogroups and 7 different H types were identified among the 22 STEC isolates, which belonged to 14 serotypes: O2:H45, O8:H2, O8:H16, O49:H21, O74:H8, O81:H21, O82:H19, O96:H8, O119:H19, O120:H9, O159:H21, O163:H19, O169:H8, and O170:H8. The predominant serotypes were O8:H2, O2:H45, O74:H8, and O49:H21 of which we found four (18.18%), three (13.64%), three (13.64%) and two (9.09%) isolates, respectively. The remaining 10 serotypes were each represented by only one isolate (**Figure 1**).

### Presence of *stx* Genes and *stx* Subtypes and Other Virulence Factor Genes

Among the 22 STEC isolates, five tested positive for *stx*_1_ only, 14 for *stx*_2_ only, and three for both *stx*_1_ and *stx*_2_. All of the eight *stx*_1_ were subtyped into *stx*_1a_. Of the 17 *stx*_2_-positive STEC isolates, one was subtyped into *stx*_2a_, five were *stx*_2b_, 10 were *stx*_2d_, and one isolate carried both *stx*_2a_ and *stx*_2d_ (**Figure 1**).

All 22 STEC isolates were *eae* negative. Of the five other putative adhesin genes (*iha*, *saa*, *efa1*, *paa*, *eibG*) screened, *iha* and *saa* were present in 16 (72.73%) and 15 (68.18%) isolates, respectively, and the 15 *saa* positive STEC isolates also carried *iha*. The other three adhesin genes were not detected in any of the 22 isolates. We also screened five virulence-associated genes (*ehxA*, *katP*, *toxB*, *astA*, *subA*), two markers for HPI (*irp2* and *fyuA*), and 11 non-LEE encoded effector (*nle*) genes. Fifteen (68.18%) STEC isolates were positive for *ehxA*, fourteen (63.64%) and three (13.64%) were positive for *subA* and *astA*, respectively. All our STEC isolates were negative for *katP*, *toxB*, the HPI genes and the 11 *nle* genes (**Figure 1**).

### Antibiotic Resistance

Antimicrobial resistance was determined using 23 antibiotics. All 22 STEC isolates were resistant to penicillin G. Four and one isolates were resistant to cephalothin and streptomycin, respectively, giving resistance rates of 18.18 and 4.55%. All isolates were susceptible to the remaining 20 antibiotics (**Figure 1**).

### Pulsed-Field Gel Electrophoresis (PFGE)

The 22 non-O157 STEC isolates were analyzed by PFGE to investigate their genetic relationships. All isolates produced clear bands, and could be divided into 16 PFGE patterns (EMSX11001 to EMSX11016). An UPGMA dendrogram showed that the STEC isolates were genetically diverse with nodes linking isolates at less than 90% similarity (**Figure 1**). Four isolates (12ST452, 12ST453, 12ST454, 12ST459) from intestinal contents collected in 2012, two isolates (15ST441, 15ST444) from intestinal contents collected in 2015, and three isolates (12ST456, 15ST442, and 15ST447) from intestinal contents collected in different years (two in 2015 and one in 2012) showed identical PFGE patterns, sequence type, serotype and virulence gene profile. The two isolates (15ST443, 15ST444) obtained from a single intestinal contents sample showed different PFGE patterns, serotypes and virulence gene profiles (**Figure 1**).

### Multi-locus Sequence Typing (MLST)

A total of 12 different sequence types (STs) were identified among the 22 STEC isolates, including one novel sequence type, ST5741, resulting from a novel allele type, *icd* 592. ST1001 was the most frequent ST, represented by four isolates with identical PFGE pattern, serotype, virulence gene profile and antibiotic resistance pattern. Three STs (ST297, ST906, and ST3692) were represented by three isolates each. Among these, the ST297 isolates showed different PFGE patterns, serotypes and *stx* profiles; ST906 isolates demonstrated different PFGE patterns and *stx* profiles; while ST3692 isolates exhibited an identical PFGE pattern, serotype, virulence gene profile and antibiotic resistance pattern. ST40 was detected in two isolates with different resistance patterns, and the other seven STs were found only once. There is a good concordance between MLST and PFGE; isolates of the same ST generally showed the same or similar PFGE patterns (**Figure 1**).

## Discussion

The Qinghai-Tibetan plateau is a region where the altitude is high, the temperature is low and ultraviolet radiation is strong. Plateau pikas (*O. curzoniae*) play important roles in this ecosystem, such as recycling nutrients in soil, providing food to predators, and providing microhabitats by increasing plant richness, while their burrows provide nests for small birds and reptiles (Yu et al., 2012). Plateau pikas have been proved to be a reservoir of some pathogens (Zhang et al., 2013; Yu et al., 2014). In the current research, we found that 5.2% (58/1116) of the plateau pika samples were positive for the *stx* genes by PCR and 1.88% (21/1116) by microbiological culture. The prevalence of STEC in plateau pikas is much lower than that in yaks from the same geographical region (Bai et al., 2013), where 11.68% (85/728) of samples yielded STEC strains. Notably, more than 60% of the *stx*-positive samples by PCR were negative by culture in this study. It was inferred that several factors may result in the failure to isolate STEC from the *stx*-positive samples, such as the low levels of STEC in the samples, the perturbation of high levels of background microflora, the presence of other bacteria carrying *stx*, the interference of free Stx phages (Quiros et al., 2015). Different STEC isolation methods may also contribute to the recovery of different serotype strains (Kalchayanand et al., 2013) and dual plating on lesser and more highly selective agars may maximize the recovery of target STEC strains (Gill et al., 2014). A possible reason for no isolation of STEC O157 and the top six non-O157 STEC strains might be the method used. In this study, we used CHROMagar^TM^ ECC and MacConkey agars and didn’t specifically target O157 or the top six serogroups. For instance, use of O157 or the top six serogruops specific immunomagnetic beads to capture the bacteria from enrichment broth with subsequent culture in selective media are required in further study to improve the isolation of these strains. Notably, STEC were similarly distributed in intestine content samples collected in the years 2012 and 2015, while the prevalence in feces was much lower, according with observations on swine STEC strains in our previous study (Meng et al., 2014).

The STEC isolates from plateau pikas were highly genetically diverse, revealed by serotyping, PFGE, MLST, and virulence gene profiles. Isolates from different samples collected in the same or different years (four isolates from year 2012, two isolates from 2015, and three isolates from 2012 and 2015) had an identical PFGE pattern, sequence type, serotype and virulence gene profile, implying some STEC clones may be widespread and persistent in this region. STEC isolates 15ST443 and 15ST444 obtained from the same intestinal contents sample showed different PFGE patterns, serotypes and virulence gene profiles, indicating that some plateau pikas could be colonized by more than one STEC clone.

The predominant serotypes O8:H2 and O2:H45 were also observed in yak STEC isolates, and O2:H45 was the most common serotype among yak isolates (Bai et al., 2013). Nine sequence types (ST40, ST297, ST737, ST906, ST1001, ST3686, ST3692, ST3883, and ST3884) were identified in STEC from both yaks (Sun et al., 2013) and plateau pikas, suggesting some STEC strains may be widely spread among animals in the same geographical region; however, the transmission route remains to be understood. Resistance to multiple antimicrobials has been shown in many non-O157 STEC isolates from humans, animals and food (Stephan and Schumacher, 2001). Wong et al. (2000) supported the theory that antibiotics play an important role in the progression of HUS and therefore the resistances of STEC may have relevance to clinical symptoms. In the present study, 20 of the 23 antimicrobial agents were active against all 22 plateau pika STEC isolates, the exceptions being penicillin G, cephalothin and streptomycin. These results were quite different from those observed in healthy pigs in China, where only two of the 23 antimicrobial agents (imipenem and meropenem) were active against all the STEC isolates (Meng et al., 2014). The lower antibiotic resistance may due to a low prevalence of drug resistant bacteria in this ecosystem.

**FIGURE 2:**
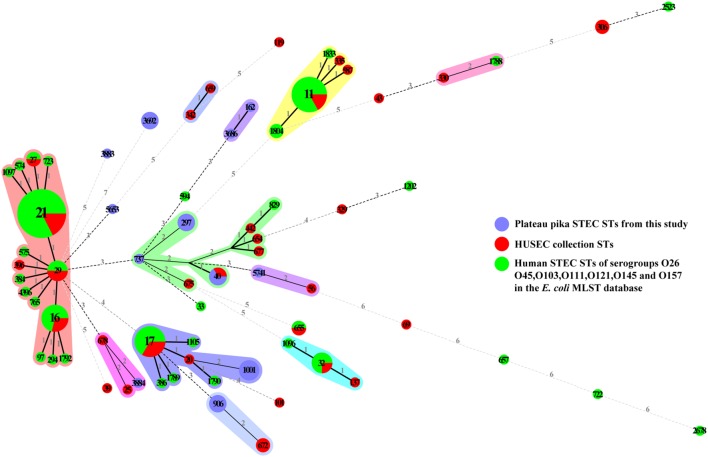
**Minimum spanning tree of 12 STs from this study (blue), 32 STs from the HUSEC collection (red), and 36 STs from human STEC STs of serogroups O26, O45, O103, O111, O121, O145, and O157 in the *E. coli* MLST database (green).** Each circle represents a ST, with the pie divided proportionally to the number of isolates in that ST from different sources. The number in a circle indicates the ST number. The numbers on connecting lines represent the number of allelic differences between two STs.

Shiga toxin (Stx) production, especially Stx1a, Stx2a, Stx2c, and Stx2d, has been implicated in causing severe disease and HUS, and STEC carrying Stx2b are mainly associated with diarrheal disease (Friedrich et al., 2002; Jelacic et al., 2003; Bielaszewska et al., 2006). Adherence to the intestinal epithelium and subsequent colonization are crucial for the STEC pathogenic process. STEC isolates that carry both *stx*_2_ and *eae* genes are often associated with severe disease (Werber et al., 2003). Coombes et al. (2008) reported that non-LEE encoded effector (*nle*) genes are correlated with outbreak and HUS potential in humans. In the present study, different profiles, i.e., *stx*_1a_, *stx*_2a_, *stx*_2b_, *stx*_2d_, *stx*_1a_ + *stx*_2d_, and *stx*_2a_ + *stx*_2d_, were present in the 22 non-O157 STEC isolates, but all were *eae* negative. Some of the isolates carried virulence genes *ehxA* (68.18%), *subA* (63.64%), *astA* (13.64%), or adherence genes *iha* (72.23%) and *saa* (68.18%). Other novel virulence or adherence factors in these STEC isolates besides *stx* need to be further investigated. Multi-locus sequence typing identified 12 STs obtained in this study. The 32 STs of the HUSEC collection and 36 human STEC STs from the *E. coli* MLST database were considered to assess the potential risk of human infection by plateau pika STEC strains. None of the STs of plateau pika STEC were identical to any of the human STEC STs. Only one, ST40, with two isolates (15ST441 and 15ST444), was also observed in the HUSEC collection (**Figure 2**), but these two isolates harbored the low-virulence *stx*_2b_ gene and no other virulence genes were detected. These data suggest that STEC in plateau pikas may have low potential to cause human disease.

In China, STEC O157 strains were detected in different animals and caused a major outbreak in 1999 (Xiong et al., 2012; Meng et al., 2013). Non-O157 STEC strains have been isolated from different animals and raw meats in China as reported in our previous studies (Bai et al., 2013, 2015; Meng et al., 2014). In recent years, STEC isolates including O157 and non-O157 serogroups have been reported in diarrheal patient by several other studies in China (Chen et al., 2014; Wang et al., 2015; Yu et al., 2015), but limited information is available regarding the phenotypic and genetic characteristics of these STEC isolates. Thus, further indepth studies on STECs from different resources in China are required to determine their pathogenic potential.

## Conclusion

We initially revealed that plateau pikas may be a natural reservoir of STEC, extending our knowledge of the genetic diversity and reservoir host range of STEC. Based on comparison by serotypes and MLST analysis with human strains and presence of virulence genes, the majority of these pika STECs may have a low potential to cause human disease. However, further investigations are needed to assess the public health significance in Tibetans and nomadic pastoralists in this geographic region.

## Author Contributions

XB, JX, YX, and SL designed the project, analyzed data and wrote the manuscript. XT and YX collected samples. WZ, YX, HS, XL, and JP carried out the experiments.

## Conflict of Interest Statement

The authors declare that the research was conducted in the absence of any commercial or financial relationships that could be construed as a potential conflict of interest.

## Footnotes

1 http://cge.cbs.dtu.dk/services/

2 http://www.cdc.gov/pulsenet/pathogens/index.html

3 http://mlst.warwick.ac.uk/mlst/dbs/Ecoli
