# Supplementary material for: Shiga Toxin-Producing Escherichia coli in Plateau Pika (Ochotona curzoniae) on the Qinghai-Tibetan Plateau, China
Source: Front Microbiol. 2016 Mar 22;7:375. doi: 10.3389/fmicb.2016.00375 (PMC4802371; doi:10.3389/fmicb.2016.00375)
Supplement: Supplementary file 1 [file Table_1.DOC]

**TABLE S1. PCR primers used for the detection of STEC virulence or adherence genes**

| Target | Primer forward/Primer reverse (5′-3′) | Amplicon size (bp) | Annealing temperature (°C) | Reference |
| --- | --- | --- | --- | --- |
| *stx*_1_ | AAATCGCCATTCGTTGACTACTTCT/ | 370 | 58 | a |
|  | TGCCATTCTGGCAACTCGCGATGCA |  |  |
| *stx*_2_ | CAGTCGTCACTCACTGGTTTCATCA/ | 283 | 58 | a |
|  | GGATATTCTCCCCACTCTGACACC |  |  |
| *stx*_2_ | ATGAAGTGTATATTATTTAAATGG/ | 1260 | 55 | b |
|  | TCAGTCATTATTAAACTGCAC |  |  |
| *eae* | TCAATGCAGTTCCGTTATCAGTT/ | 482 | 58 |  |
|  | GTAAAGTCCGTTACCCCAACCTG |  |  |
| *iha* | CAGTTCAGTTTCGCATTCACC/ | 1305 | 56 |  |
|  | GTATGGCTCTGATGCGATG |  |  |
| *efa1* | GAGACTGCCAGAGAAAG/ | 479 | 51 |  |
|  | GGTATTGTTGCATGTTCAG |  |  |
| *saa* | CGTGATGAACAGGCTATTGC/ | 119 | 52 |  |
|  | ATGGACATGCCTGTGGCAAC |  |  |
| *paa* | ATGAGGAAACATAATGGCAGG/ | 350 | 60 |  |
|  | TCTGGTCAGGTCGTCAATAC |  |  |
| *eibG* | ATCGGCTTTCATCGCATCAGGAC/ | 547 | 60 |  |
|  | CCACAAGGCGGGTATTCGTATC |  |  |
| *astA* | CCATCAACACAGTATATCCGA/ | 111 | 55 |  |
|  | GGTCGCGAGTGACGGCTTTGT |  |  |
| *subA* | TATGGCTTCCCTCATTGCC/ | 556 | 65/60 |  |
|  | TATAGCTGTTGCTTCTGACG |  |  |
| *ehxA* | GGTGCAGCAGAAAAAGTTGTAG/ | 1551 | 57 |  |
|  | TCTCGCCTGATAGTGTTTGGTA |  |  |
| *katP* | CTTCCTGTTCTGATTCTTCTGG/ | 2125 | 56 |  |
|  | AACTTATTTCTCGCATCATCC |  |  |
| *toxB* | ATACCTACCTGCTCTGGATTGA/ | 602 | 55 |  |
|  | TTCTTACCTGATCTGATGCAGC |  |  |  |
| *fyuA* | TGATTAACCCCGCGACGGGAA/ | 880 | 57 |  |
|  | CGCAGTAGGCACGATGTTGTA |  |  |  |
| *irp2* | AAGGATTCGCTGTTACCGGAC/ | 280 | 54 |  |
|  | TCGTCGGGCAGCGTTTCTTCT |  |  |  |
| *ent* | GAATAACAATCACTCCTCACC/ | 233 | 55 |  |
|  | TTACAGTGCCCGATTACG |  |  |
| *nleA* | ATGAACATTCAACCGACCATAC/ | 1296 | 55 |  |
|  | GACTCTTGTTTCTTGGATTATATCAAA |  |  |
| *nleB* | GGAAGTTTGTTTACAGAGACG/ | 297 | 55 |  |
|  | AAAATGCCGCTTGATACC |  |  |
| *nleB2* | GTTAATACTAAGCAGCATCC/ | 475 | 52 |  |
|  | CCATATCAAGATAGATACACC |  |  |
| *nleC* | ACAGTCCAACTTCAACTTTTCC/ | 777 | 55 |  |
|  | ATCGTACCCAGCCTTTCG |  |  |
| *nleD* | GGTATTACATCAGTCATCAAGG/ | 426 | 55 |  |
|  | TTGTGGAAAACATGGAGC |  |  |
| *nleE* | GTATAACCAGAGGAGTAGC/ | 260 | 52 |  |
|  | GATCTTACAACAAATGTCC |  |  |
| *nleF* | ATGTTACCAACAAGTGGTTCTTC/ | 567 | 55 |  |
|  | ATCCACATTGTAAAGATCCTTTGTT |  |  |
| *nleG* | ATGTTATCGCCCTCTTCTATAAAT/ | 906 | 55 |  |
|  | ACTTAATACTACACTAATAAGATCCA |  |  |
| *nleH1-1* | GTTACCACCTTAAGTATCC/ | 456 | 55 |  |
|  | GTTTCTCATGAACACTCC |  |  |
| *nleH1-2* | AACGCCTTATATTTTACC/ | 589 | 52 |  |
|  | AGCACAATTATCTCTTCC |  |  |

^a^ Primers used for duplex PCR for the detection of *stx*_1_ and *stx*_2_.

^b^ Primers used for amplifying and sequencing the full length of *stx*_2_.

**REFERENCES**

Brian, M.J., Frosolono, M., Murray, B.E., Miranda, A., Lopez, E.L., Gomez, H.F., et al. (1992). Polymerase chain reaction for diagnosis of enterohemorrhagic *Escherichia coli* infection and hemolytic-uremic syndrome. *J .Clin .Microbiol.* 30**,** 1801-1806. doi: 10.1099/13500872-142-11-3305

Brunder, W., Schmidt, H., and Karch, H. (1996). KatP, a novel catalase-peroxidase encoded by the large plasmid of enterohaemorrhagic *Escherichia coli* O157:H7. *Microbiology.* 142**,** 3305-3315. doi: 10.1128/AEM.02566-07

Coombes, B.K., Wickham, M.E., Mascarenhas, M., Gruenheid, S., Finlay, B.B., and Karmali, M.A. (2008). Molecular analysis as an aid to assess the public health risk of non-O157 Shiga toxin-producing *Escherichia coli* strains. *Appl. Environ. Microbiol.* 74**,** 2153-2160.

Gunzer, F., Bohm, H., Russmann, H., Bitzan, M., Aleksic, S., and Karch, H. (1992). Molecular detection of sorbitol-fermenting *Escherichia coli* O157 in patients with hemolytic-uremic syndrome. *J. Clin. Microbiol.* 30**,** 1807-1810.

Karch, H., Schubert, S., Zhang, D., Zhang, W., Schmidt, H., Olschlager, T., et al. (1999). A genomic island, termed high-pathogenicity island, is present in certain non-O157 Shiga toxin-producing *Escherichia coli* clonal lineages. *Infect. Immun.* 67**,** 5994-6001.

Lu, Y., Iyoda, S., Satou, H., Itoh, K., Saitoh, T., and Watanabe, H. (2006). A new immunoglobulin-binding protein, EibG, is responsible for the chain-like adhesion phenotype of locus of enterocyte effacement-negative, shiga toxin-producing *Escherichia coli*. *Infect. Immun.* 74**,** 5747-5755. doi: 10.1128/IAI.00724-06

Nicholls, L., Grant, T.H., and Robins-Browne, R.M. (2000). Identification of a novel genetic locus that is required for in vitro adhesion of a clinical isolate of enterohaemorrhagic *Escherichia coli* to epithelial cells. *Mol. Microbiol.* 35**,** 275-288.

Paton, A.W., and Paton, J.C. (2002). Direct detection and characterization of Shiga toxigenic *Escherichia coli* by multiplex PCR for *stx1*, *stx2*, *eae*, *ehxA*, and *saa*. *J. Clin. Microbiol.* 40**,** 271-274.

Paton, A.W., and Paton, J.C. (2005). Multiplex PCR for direct detection of Shiga toxigenic *Escherichia coli* strains producing the novel subtilase cytotoxin. *J. Clin. Microbiol.* 43**,** 2944-2947. doi: 10.1128/JCM.43.6.2944-2947.2005

Schmidt, H., Beutin, L., and Karch, H. (1995). Molecular analysis of the plasmid-encoded hemolysin of *Escherichia coli* O157:H7 strain EDL 933. *Infect. Immun.* 63**,** 1055-1061.

Schmidt, H., Zhang, W.L., Hemmrich, U., Jelacic, S., Brunder, W., Tarr, P.I., et al. (2001). Identification and characterization of a novel genomic island integrated at *selC* in locus of enterocyte effacement-negative, Shiga toxin-producing *Escherichia coli*. *Infect. Immun.* 69**,** 6863-6873. doi: 10.1128/IAI.69.11.6863-6873.2001

Tarr, C.L., Large, T.M., Moeller, C.L., Lacher, D.W., Tarr, P.I., Acheson, D.W., et al. (2002). Molecular characterization of a serotype O121:H19 clone, a distinct Shiga toxin-producing clone of pathogenic *Escherichia coli*. *Infect. Immun.* 70**,** 6853-6859.

Yamamoto, T., and Echeverria, P. (1996). Detection of the enteroaggregative *Escherichia coli* heat-stable enterotoxin 1 gene sequences in enterotoxigenic *E. coli* strains pathogenic for humans. *Infect. Immun.* 64**,** 1441-1445.

Zweifel, C., Schumacher, S., Beutin, L., Blanco, J., and Stephan, R. (2006). Virulence profiles of Shiga toxin 2e-producing *Escherichia coli* isolated from healthy pig at slaughter. *Vet. Microbiol.* 117**,** 328-332. doi: 10.1016/j.vetmic.2006.06.017
